# *Ap2s1* mutation causes hypercalcaemia in mice and impairs interaction between calcium-sensing receptor and adaptor protein-2

**DOI:** 10.1093/hmg/ddab076

**Published:** 2021-03-17

**Authors:** Fadil M Hannan, Mark Stevenson, Asha L Bayliss, Victoria J Stokes, Michelle Stewart, Kreepa G Kooblall, Caroline M Gorvin, Gemma Codner, Lydia Teboul, Sara Wells, Rajesh V Thakker

**Affiliations:** 1 Academic Endocrine Unit, Radcliffe Department of Medicine, University of Oxford, Oxford OX3 7LJ, UK; 2 Nuffield Department of Women’s and Reproductive Health, University of Oxford, Oxford OX3 9DU, UK; 3 Mammalian Genetics Unit and Mary Lyon Centre, MRC Harwell Institute, Harwell Campus, Oxfordshire OX11 0RD, UK

## Abstract

Adaptor protein 2 (AP2), a heterotetrameric complex comprising AP2α, AP2β2, AP2μ2 and AP2σ2 subunits, is ubiquitously expressed and involved in endocytosis and trafficking of membrane proteins, such as the calcium-sensing receptor (CaSR), a G-protein coupled receptor that signals via Gα_11_. Mutations of CaSR, Gα_11_ and AP2σ2, encoded by *AP2S1,* cause familial hypocalciuric hypercalcaemia types 1–3 (FHH1–3), respectively. FHH3 patients have heterozygous *AP2S1* missense Arg15 mutations (p.Arg15Cys, p.Arg15His or p.Arg15Leu) with hypercalcaemia, which may be marked and symptomatic, and occasional hypophosphataemia and osteomalacia. To further characterize the phenotypic spectrum and calcitropic pathophysiology of FHH3, we used CRISPR/Cas9 genome editing to generate mice harboring the *AP2S1* p.Arg15Leu mutation, which causes the most severe FHH3 phenotype. Heterozygous (*Ap2s1^+/L15^*) mice were viable, and had marked hypercalcaemia, hypermagnesaemia, hypophosphataemia, and increases in alkaline phosphatase activity and fibroblast growth factor-23. Plasma 1,25-dihydroxyvitamin D was normal, and no alterations in bone mineral density or bone turnover were noted. Homozygous (*Ap2s1^L15/L15^*) mice invariably died perinatally. Co-immunoprecipitation studies showed that the *AP2S1* p.Arg15Leu mutation impaired protein–protein interactions between AP2σ2 and the other AP2 subunits, and also with the CaSR. Cinacalcet, a CaSR positive allosteric modulator, decreased plasma calcium and parathyroid hormone concentrations in *Ap2s1^+/L15^* mice, but had no effect on the diminished AP2σ2-CaSR interaction *in vitro*. Thus, our studies have established a mouse model that is representative for FHH3 in humans, and demonstrated that the *AP2S1* p.Arg15Leu mutation causes a predominantly calcitropic phenotype, which can be ameliorated by treatment with cinacalcet.

## Introduction

Familial hypocalciuric hypercalcaemia (FHH) is an autosomal dominant disorder of extracellular calcium metabolism characterized by lifelong increases of serum calcium concentrations, mild hypermagnesaemia, normal or elevated circulating parathyroid hormone (PTH) concentrations, and inappropriately low urinary calcium excretion (urine calcium to creatinine clearance ratio (CCCR) < 0.01) (1). FHH is a genetically heterogeneous disorder comprising three reported variants. FHH types 1 and 2 (FHH1, OMIM #145980; FHH2, OMIM #145981) are generally associated with mild asymptomatic hypercalcaemia and caused by loss-of-function mutations of the calcium-sensing receptor (CaSR), a G-protein coupled receptor (GPCR), and G-protein subunit α_11_ (Gα_11_), respectively, which are pivotal for regulating PTH secretion and renal tubular calcium reabsorption (2). In contrast, FHH type 3 (FHH3, OMIM #600740) is associated with a more severe biochemical phenotype that is characterized by significantly higher serum calcium and magnesium concentrations and a significantly reduced CCCR, when compared with FHH1 (3). Furthermore, FHH3 may be associated with symptomatic hypercalcaemia and reduced bone mineral density (BMD), and occasionally also osteomalacia as well as neurodevelopmental disorders (3–6). FHH3 is caused by germline heterozygous loss-of-function mutations of the *AP2S1* gene, which is located on chromosome 19q13.3 and encodes the AP2σ2 protein (7). *AP2S1* mutations have been reported in ~70 FHH probands to-date, and affected individuals harbor a mutation affecting the AP2σ2 Arg15 residue, which may give rise to a p.Arg15Cys, p.Arg15His or p.Arg15Leu missense mutation (3,5–11). FHH3 patients harboring the p.Arg15Leu AP2σ2 mutation have been reported to have greater hypercalcaemia and to present at an earlier age than probands with p.Arg15Cys or p.Arg15His AP2σ2 mutations (3).

The AP2σ2 protein is evolutionarily highly conserved (7), and forms part of the ubiquitously expressed heterotetrameric adaptor protein-2 (AP2) complex, which also comprises AP2α, AP2β2 and AP2μ2 subunits (12). The AP2 complex plays a pivotal role in clathrin-mediated endocytosis by initiating the formation of clathrin-coated vesicles, which leads to trafficking of plasma membrane constituents to endosomes (13,14). AP2σ2 contributes to the AP2 core structure (15), which binds to transmembrane cargo proteins such as GPCRs. Consistent with this, AP2σ2 has been shown to regulate CaSR endocytosis, and the FHH3-causing p.Arg15Cys, p.Arg15His and p.Arg15Leu mutations have all been demonstrated to impair CaSR endocytosis, thereby decreasing signaling from the endosomal CaSR (16).

We have sought to establish a mouse model to: facilitate investigation of the *in vivo* roles of the AP2σ2 protein; further characterize the calcitropic phenotype and pathophysiology of FHH3; and evaluate CaSR-targeted therapy for this disorder. Mice harboring the AP2σ2 p.Arg15Leu mutation were generated, as this is associated with the clinically most severe phenotype in FHH3 patients.

## Results

### Generation of mice harboring an Ap2s1 mutation, p.Arg15Leu

Mutant mice on a C57BL/6 J strain background were generated using CRISPR/Cas9 genome editing, as reported in (17). Founder mice harbored a G-to-T transversion at c.44 within exon 2 of the *Ap2s1* gene, which was predicted to lead to a missense substitution of Arg, encoded by CGC, to Leu, encoded by CTC, at *Ap2s1* codon 15 (Supplementary Material, Fig. S1). F1 generation mice were shown to harbor WT (Arg15) and mutant (Leu15) *Ap2s1* alleles, and mice derived from intercrosses of heterozygous mutant mice showed the expected Mendelian inheritance ratio of 1:2:1 at birth for the WT (*Ap2s1^+/+^*), heterozygous (*Ap2s1^+/L15^*) and homozygous (*Ap2s1^L15/L15^*) genotypes, respectively, which were confirmed by DNA sequence analysis (Table 1, Supplementary Material, Fig. S1). WT and *Ap2s1^+/L15^* mice were viable and survived into adulthood (Table 1). However, >85% of *Ap2s1^L15/L15^* mice did not survive into adulthood (Table 1), and most died within 48 h after birth. Because of the high rate of homozygote neonatal lethality, WT and *Ap2s1^+/L15^* mice were generated for subsequent studies by backcrossing *Ap2s1^+/L15^* mice onto the WT C57BL/6 J strain background.

**Table 1:** Proportion of offspring bred from crosses of *Ap2s1^+/L15^ × Ap2s1^+/L15^* mice

Genotype	Number of offspring observed at birth (*n* = 72)	Number of offspring observed at weaning (~3 weeks old) (*n* = 48)	Number of offspring observed at adulthood (>10 weeks old) (*n* = 46)
*+/+* (WT)	17 (24%)	14 (29%)	14 (31%)
*+/L15* (Het)	38 (52%)	30 (63%)	30 (65%)
*L15/L15* (Hom)	17 (24%)	4 (8%)^**^	2 (4%)^***^

WT, wild type; Het, heterozygotes; Hom, homozygotes. Heterozygous crosses led to the expected Mendelian inheritance ratio of 1:2:1 for WT: Het: Hom offspring at birth. However, the observed ratio of offspring genotypes at weaning and adulthood was significantly different from the expected ratio, due to homozygote mice having reduced viability beyond the perinatal period. ^**^P < 0.01 and ^***^P < 0.001 (by binomial distribution analysis).

### Phenotype of mice harboring the Ap2s1 mutation, p.Arg15Leu

**Figure 1:**
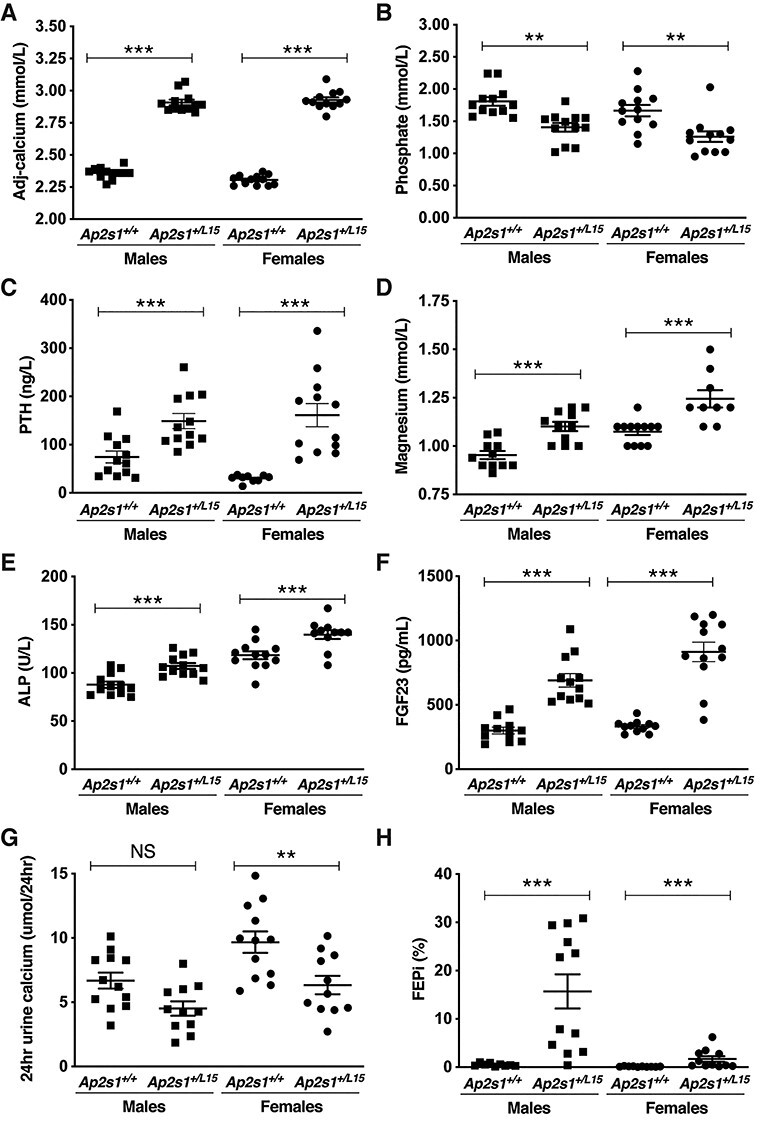
Calcitropic phenotype of adult WT (*Ap2s1^+/+^*) and *Ap2s1^+/L15^* mice, aged 15–17 weeks. (**A**–**F**) Plasma calcitropic phenotype: (A) adjusted-calcium; (B) phosphate; (C) PTH; (D) magnesium; (E) ALP and (F) fibroblast growth factor-23 (FGF23). (**G** and **H**) Urinary calcitropic phenotype: (G) 24 h urine calcium; (H) FEPi. Mean ± SEM values for respective groups (*n* = 7–12 mice per group) are indicated. NS, non-significant; ^*^^*^P < 0.01; ^*^^*^^*^P < 0.001 for *Ap2s1^+/L15^* mice versus respective WT mice. One-way ANOVA followed by Sidak’s test for pairwise multiple comparisons were used for all analyses.

Adult *Ap2s1^+/L15^* mice, aged 12–22 weeks, showed no gross morphological abnormalities, although male *Ap2s1^+/L15^* mice had a significantly reduced body weight when compared with age-matched WT male litter-mates, whereas female *Ap2s1^+/L15^* mice had a normal body weight (Table 2). Activities such as eating, drinking, grooming, moving and interacting with cage-mates were assessed qualitatively by visual inspection of mice in their home cages, and observed to be similar between *Ap2s1^+/L15^* mice and their WT littermates. Plasma biochemical analysis showed that male and female *Ap2s1^+/L15^* mice had substantial hypercalcaemia with mean calcium concentrations >10 SD above that of respective WT mice (Table 2, Fig. 1A). This was associated with hypophosphataemia, and significant increases in plasma PTH, magnesium and alkaline phosphatase (ALP) activity (Table 2, Fig. 1B-E). Male and female *Ap2s1^+/L15^* mice also showed marked increases in plasma fibroblast growth factor-23 (FGF23), which were not associated with any significant alterations in plasma 1,25-dihydroxyvitamin D concentrations (Table 2, Fig. 1F). Urine biochemical analysis showed that female *Ap2s1^+/L15^* mice had significantly reduced 24 h urine calcium excretion, whereas male and female *Ap2s1^+/L15^* mice showed a significantly increased fractional excretion of phosphate (FEPi) (Table 2, Fig. 1G and H). Bone metabolism was assessed by whole body dual-energy X-ray absorptiometry (DXA), and by measurement of the pro collagen type 1 N-terminal pro-peptide (P1NP) and C-terminal cross-linking telopeptide of type 1 collagen (CTX-1) bone turnover markers, as reported (18,19). Bone mineral content (BMC) corrected for body weight, BMD, and bone turnover in male and female *Ap2s1^+/L15^* mice were not significantly different to those observed in age- and sex-matched WT mice (Table 2 and Supplementary Material, Table S1).

**Table 2:** Age, body weight and calcitropic biochemical parameters of adult WT (*+/+*) and *Ap2s1^+/L15^* (*+/L15*) mice, aged 15–17 weeks

	Male		Female	
	*+/+*	*+/L15*	*+/+*	*+/L15*
Age (weeks)	16.5 ± 0.3 (*n* = 12)	16.4 ± 0.3 (*n* = 12)	16.4 ± 0.3 (*n* = 12)	16.3 ± 0.2 (*n* = 12)
Weight (g)	30 ± 0.3 (*n* = 12)	28.4 ± 0.5 (*n* = 12)^*^	23.9 ± 0.4 (*n* = 12)	22.7 ± 0.5 (*n* = 12)
*Plasma biochemistry*				
Adj-calcium (mmol/l)^a^	2.36 ± 0.01 (*n* = 12)	2.91 ± 0.02 (*n* = 12)^***^	2.31 ± 0.01 (*n* = 12)	2.93 ± 0.02 (*n* = 12)^***^
PTH (ng/l)	74.4 ± 12.2 (*n* = 12)	149 ± 16 (*n* = 12)^***^	29.9 ± 2.4 (*n* = 9)	161 ± 24 (*n* = 12)^***^
Phosphate (mmol/l)	1.81 ± 0.07 (*n* = 12)	1.41 ± 0.07 (*n* = 12)^**^	1.67 ± 0.1 (*n* = 12)	1.26 ± 0.08 (*n* = 12)^**^
Magnesium (mmol/l)	0.95 ± 0.02 (*n* = 11)	1.10 ± 0.02 (*n* = 11)^***^	1.08 ± 0.02 (*n* = 12)	1.24 ± 0.04 (*n* = 9)^***^
ALP (U/l)	87.8 ± 3.3 (*n* = 12)	107 ± 3.1 (*n* = 12)^***^	118 ± 4.2 (*n* = 12)	143 ± 3.7 (*n* = 10)^***^
1,25D (pmol/l)^b^	101 ± 11 (*n* = 12)	113 ± 7 (*n* = 12)^†^	76.1 ± 6.5 (*n* = 8)	106 ± 11 (*n* = 7)^††^
FGF23 (ng/l)	301 ± 26 (*n* = 11)	691 ± 53 (*n* = 12)^***^	332 ± 14 (*n* = 11)	912 ± 75 (*n* = 12)^***^
P1NP (μg/l)	60.7 ± 4.8 (*n* = 11)	63.7 ± 5.9 (*n* = 11)	36.4 ± 1.6 (*n* = 12)	41.0 ± 2.2 (*n* = 12)
CTX-1 (μg/l)	0.47 ± 0.1 (*n* = 8)	0.34 ± 0.07 (*n* = 9)	0.07 ± 0.02 (*n* = 4)	0.03 ± 0.01 (*n* = 7)^†††^
*Urine biochemistry*				
24 h urine vol. (mL)	5.3 ± 0.2 (*n* = 12)	5.5 ± 0.2 (*n* = 12)	5.2 ± 0.3 (*n* = 12)	5.8 ± 0.2 (*n* = 12)
24 h calcium (μmol/24 h)	6.68 ± 0.6 (*n* = 12)	4.51 ± 0.6 (*n* = 11)	9.67 ± 0.8 (*n* = 12)	6.34 ± 0.7 (*n* = 11)^**^
FECa (%)	0.13 ± 0.01 (*n* = 12)	0.1 ± 0.01 (*n* = 11)	0.21 ± 0.02 (*n* = 12)	0.15 ± 0.2 (*n* = 11)^$^
FEPi (%)	0.48 ± 0.1 (*n* = 9)	15.7 ± 3.5 (*n* = 12)^***^	0.11 ± 0.02 (*n* = 10)	1.7 ± 0.59 (*n* = 11)^***^

ALP, alkaline phosphatase activity; CTX-1, C-terminal cross-linking telopeptide of type 1 collagen; FECa; fractional excretion of calcium; FEPi; fractional excretion of phosphate; P1NP, pro collagen type 1 N-terminal pro peptide; PTH, parathyroid hormone; 1,25D, 1,25-dihydroxyvitamin D; FGF23, fibroblast growth factor-23. All values are expressed as mean ± SEM. ^$^*P* = 0.05; ^*^P < 0.05; ^**^P < 0.01, ^***^P < 0.001, for *+/L15* mice versus respective WT mice. For groups with sample sizes of *n* ≤ 8, the *P*-values for *+/L15* mice versus respective WT mice are as follows: ^†^*P* = 0.68; ^††^*P* = 0.19; ^†††^*P* = 0.17. One-way ANOVA followed by Sidak’s test for pairwise multiple comparisons were used for all analyses.

^a^Plasma calcium concentrations were adjusted for the plasma albumin concentration.

^b^1,25D was measured in a separate cohort of age-matched mice.

FHH3 patients have been reported to have age-related increases in PTH concentrations (4), and we therefore assessed plasma PTH concentrations in young WT and *Ap2s1^+/L15^* mice aged 8 weeks, and also when they were mature adult mice aged 16 weeks. This analysis demonstrated an age-related increase in plasma PTH for female *Ap2s1^+/L15^* mice, which was not observed in female WT mice, or in male WT or mutant mice (Supplementary Material, Table S2). This age-related increase in plasma PTH in female *Ap2s1^+/L15^* mice was not associated with alterations in plasma calcium or phosphate concentrations (Supplementary Material, Table S2).

Non-calcitropic abnormalities were observed in female *Ap2s1^+/L15^* mice, only. Thus, female *Ap2s1^+/L15^* mice had significant decreases in plasma urea and cholesterol, and a significant increase in plasma bilirubin (Supplementary Material, Table S3). These alterations in non-calcitropic biochemical parameters were not observed in male *Ap2s1^+/L15^* mice (Supplementary Material, Table S3).

Two male homozygous (*Ap2s1^L15/L15^*) mice survived into adulthood, and these were found to have plasma calcium concentrations > 10 SD and > 5 SD above the mean values of age-matched male WT and *Ap2s1^+/L15^* mice, respectively (Supplementary Material, Table S4).

### Effect of cinacalcet on the hypercalcaemia of mice harboring the Ap2s1 mutation, p.Arg15Leu

**Figure 2:**
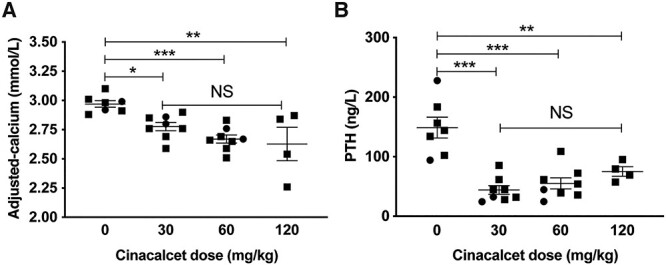
Cinacalcet dose-ranging study in adult *Ap2s1^+/L15^* mice aged 14–18 weeks. Effect of 0, 30, 60 and 120 mg/kg doses of cinacalcet on: (**A**) plasma albumin-adjusted calcium measured at 120 min post-dose; and (**B**) PTH measured at 30 min post-dose. Mean ± SEM values for respective groups (*n* = 4–8 *Ap2s1^+/L15^* mice per group) are indicated. Squares, males; circles, females. NS, non-significant; ^*^P < 0.05; ^*^^*^P < 0.01; ^*^^*^^*^P < 0.001 for cinacalcet-treated *Ap2s1^+/L15^* mice versus vehicle-treated *Ap2s1^+/L15^* mice. One-way ANOVA followed by Sidak’s test for pairwise multiple comparisons were used for all analyses.

**Figure 3:**
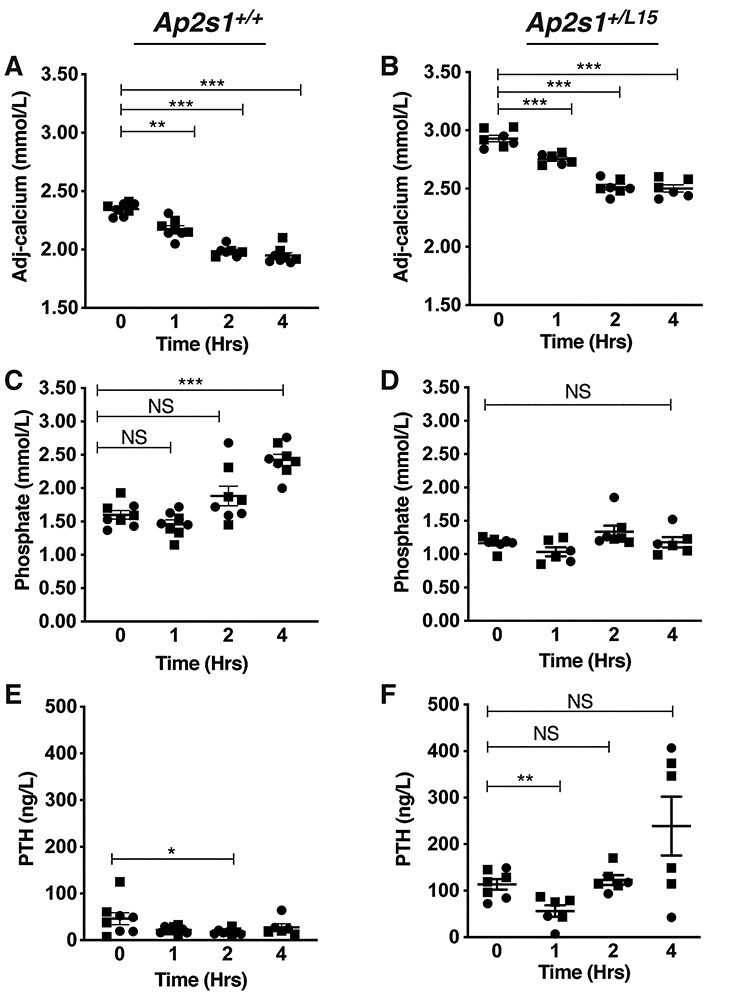
Effect of 60 mg/kg cinacalcet on plasma calcium, phosphate and parathyroid hormone at 0, 1, 2 and 4 h post dose in adult WT (*Ap2s1^+/+^*) and *Ap2s1^+/L15^* mice, aged 15–22 weeks. (**A** and **B**) plasma albumin-adjusted calcium; (**C** and **D**) plasma phosphate and (**E** and **F**) PTH in *Ap2s1^+/+^* mice and *Ap2s1^+/L15^* mice, respectively. Mean ± SEM values for respective groups (*n* = 6–8 mice per group) are indicated. Squares, males; circles, females. NS, non-significant; ^*^P < 0.05; ^*^^*^P < 0.01; ^*^^*^^*^P < 0.001 for cinacalcet-treated mice versus respective untreated mice. One-way ANOVA followed by Sidak’s test for pairwise multiple comparisons were used for all analyses.

Cinacalcet, which is a CaSR positive allosteric modulator, and also known as a calcimimetic (20), has been reported to rectify impaired CaSR signaling due to FHH3-causing *AP2S1* mutations (10), and to decrease serum calcium concentrations in three FHH3 patients harboring the *AP2S1* p.Arg15Leu mutation (10,21,22). To ascertain the dose-dependent effects as well as the immediate and later actions of cinacalcet on the hypercalcaemia of FHH3, we administered single oral bolus doses of 0, 30, 60 and 120 mg/kg cinacalcet to *Ap2s1^+/L15^* mice. In rodents, plasma PTH generally decreases rapidly (within 15–60 min) following calcimimetic administration, and was measured at 30 min post-dose in this study; whereas plasma calcium was measured at 2 h post-dose, as this mineral parameter shows a maximal reduction at 1–4 h following calcimimetic treatment (23). All cinacalcet doses significantly decreased plasma concentrations of PTH and calcium compared with mice given drug vehicle alone (Fig. 2), and dose-dependent effects were not observed. We next treated WT and *Ap2s1^+/L15^* mice with a single 60 mg/kg cinacalcet bolus and monitored the effects on plasma calcium, phosphate, and PTH at 0, 1, 2 and 4 h post-dose (Fig. 3). Cinacalcet caused significant decreases in plasma calcium in WT and *Ap2s1^+/L15^* mice at 1 h post-dose, and a further reduction in calcium was observed at 2 and 4 h post-dose (Fig. 3A and B). Cinacalcet treatment caused WT mice to become hyperphosphataemic, but such alterations in plasma phosphate were not observed in *Ap2s1^+/L15^* mice (Fig. 3C and D). The 60 mg/kg cinacalcet dose significantly decreased plasma PTH concentrations in WT and *Ap2s1^+/L15^* mice at 2 and 1 h post-dose, respectively (Fig. 3E and F).

### Effect of the AP2S1 p.Arg15Leu mutation on the interaction between AP2σ2 and CaSR

**Figure 4:**
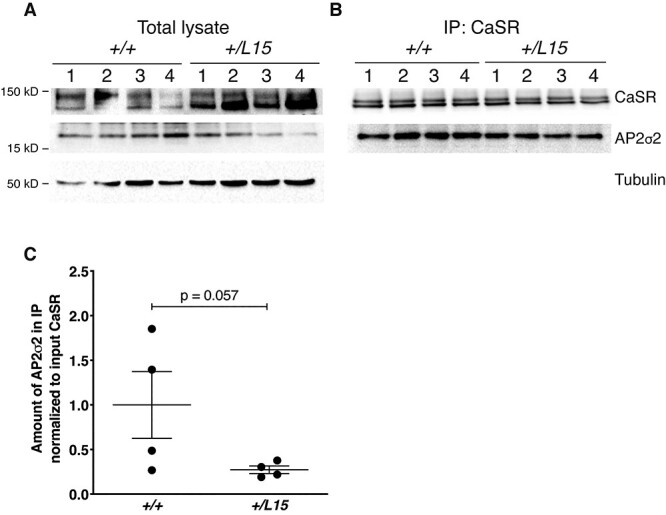
Co-immunoprecipitation analysis of the AP2σ2–CaSR interaction in *Ap2s1^+/+^* (*+/+*) and *Ap2s1^+/L15^ (+/L15*) mouse kidneys. (**A**) Immunoprecipitation using an anti-CaSR antibody and kidney cortex lysates from *n* = 4 *Ap2s1^+/+^* and *n* = 4 *Ap2s1^+/L15^* female mice (numbered 1–4 in A and B). The amount of protein in (A) the total lysates and (**B**) the precipitated immune complexes (IP:CaSR) was analyzed by western blotting using anti-CASR, anti-AP2σ2 and anti-tubulin antibodies. (**C**) Densitometry of western blots to quantify AP2σ2 in the immunoprecipitate from *Ap2s1^+/+^* and *Ap2s1^+/L15^* mice normalized to the amount of CaSR in the total lysate (pre-normalized to tubulin). Mean ± SEM values are indicated. Data were analyzed using a one-tailed Mann–Whitney *U* test.

**Figure 5:**
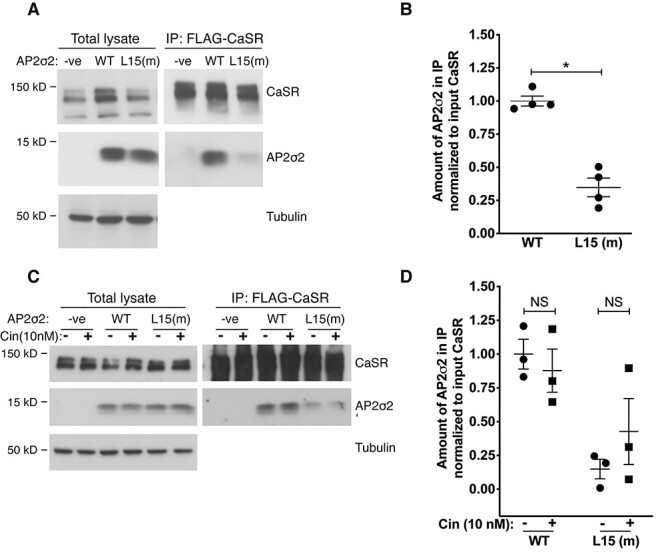
Co-immunoprecipitation analysis of the AP2σ2–CaSR interaction in HEK293 cells. (**A**) Immunoprecipitation using an anti-FLAG antibody and HEK293 cells stably expressing either: FLAG-tagged CaSR and HA-tagged WT AP2σ2 (WT); FLAG-tagged CaSR and HA-tagged mutant (m) Leu15 AP2σ2 (L15(m)); or FLAG-tagged CaSR alone (-ve). The amount of protein in the total lysates and precipitated immune complexes (IP:FLAG-CaSR) was analyzed by western blotting using anti-CASR, anti-HA and anti-tubulin antibodies. (**B**) Densitometry of western blots to quantify AP2σ2 in the immunoprecipitate of WT and L15 (m) cells normalized to the amount of CaSR in the total lysate (pre-normalized to tubulin). Mean ± SEM values are indicated and data were analyzed using the Mann–Whitney *U* test. ^*^P < 0.05. (**C**) Immunoprecipitation using an anti-FLAG antibody in -ve, WT and L15 (m) cells treated with 10 nm cinacalcet (+) and compared with vehicle-treated cells (−). The amount of protein in the total lysates and precipitated immune complexes (IP:FLAG-CaSR) was analyzed by western blotting using anti-CASR, anti-HA and anti-tubulin antibodies. (**D**) Densitometry of AP2σ2 in the immunoprecipitate from cinacalcet-treated and untreated cells. Mean ± SEM values are indicated, and data were analyzed using a using a two-way ANOVA with Bonferroni correction for multiple tests and post-hoc analysis. NS, non-significant.

FHH3-causing mutations of the AP2σ2 subunit have been shown to impair CaSR endocytosis (16), and it has been postulated that this may be because of impaired interactions between AP2σ2 and the CaSR. To investigate this, we first undertook *ex vivo* co-immunoprecipitation (co-IP) analysis using renal cortical lysates from WT and *Ap2s1^+/L15^* mice (Fig. 4). The CaSR was immunoprecipitated from lysates (Fig. 4A) using an anti-CaSR antibody, and then probed with an anti-AP2σ2 antibody. The CaSR immunoprecipitate from WT and *Ap2s1^+/L15^* mouse kidneys showed the presence of AP2σ2, thereby confirming a protein–protein interaction between the CaSR and AP2σ2 subunit (Fig. 4B). Moreover, the amount of AP2σ2 in the CaSR immunoprecipitate from *Ap2s1^+/L15^* mouse kidneys showed a >50% (*P* = 0.057) decrease compared with kidneys from WT mice, which was suggestive of a reduced interaction between the CaSR and mutant (Leu15) AP2σ2 subunit (Fig. 4C), although a limited abundance of AP2σ2 could also explain these results. We therefore further evaluated the effects of the FHH3-associated Arg15Leu AP2σ2 mutation on AP2σ2–CaSR interactions *in vitro*, by generating and using HEK293 cells that stably overexpressed an N-terminal FLAG-tagged CaSR (FlaC2 cells) with either the HA-tagged WT (Arg15) AP2σ2 subunit (Arg15–AP2σ2–FlaC2 cells) or the HA-tagged mutant (Leu15) AP2σ2 subunit (Leu15–AP2σ2–FlaC2 cells). Co-IP analysis using anti-FLAG and anti-HA antibodies showed a significant reduction of >50% in amount of AP2σ2 present in the FLAG–CaSR immunoprecipitate from mutant (Leu15–AP2σ2–FlaC2) cells compared with WT (Arg15–AP2σ2–FlaC2) cells (*P* < 0.05) (Fig. 5A and B). Thus, these studies demonstrate that the *AP2S1* p.Arg15Leu mutation diminishes the protein–protein interaction between CaSR and the AP2σ2 subunit. Cinacalcet improves the calcitropic phenotype of *Ap2s1^+/L15^* mice (Fig. 3A and B) and FHH3 patients (10,21,22), and we therefore evaluated whether this calcimimetic compound can rescue the interaction between the CaSR and mutant (Leu15) AP2σ2 subunit *in vitro*. WT (Arg15–AP2σ2–FlaC2) cells and mutant (Leu15–AP2σ2–FlaC2) cells were treated with 10 nm cinacalcet, as this dose has been reported to rectify the signaling responses of cells expressing FHH3 mutant proteins *in vitro* (10), and the AP2σ2–CaSR interaction assessed by co-IP analysis using anti-FLAG and anti-HA antibodies. Cinacalcet treatment did not alter the amount of AP2σ2 present in the FLAG–CaSR immunoprecipitates from either WT (Arg15–AP2σ2–FlaC2) cells or mutant (Leu15–AP2σ2–FlaC2) cells (Fig. 5C and D), thereby indicating that this calcimimetic does not influence the WT or mutant AP2σ2–CaSR interactions.

### Effect of the AP2S1 p.Arg15Leu mutation on AP2σ2 interactions with other AP2 complex subunits

**Figure 6:**
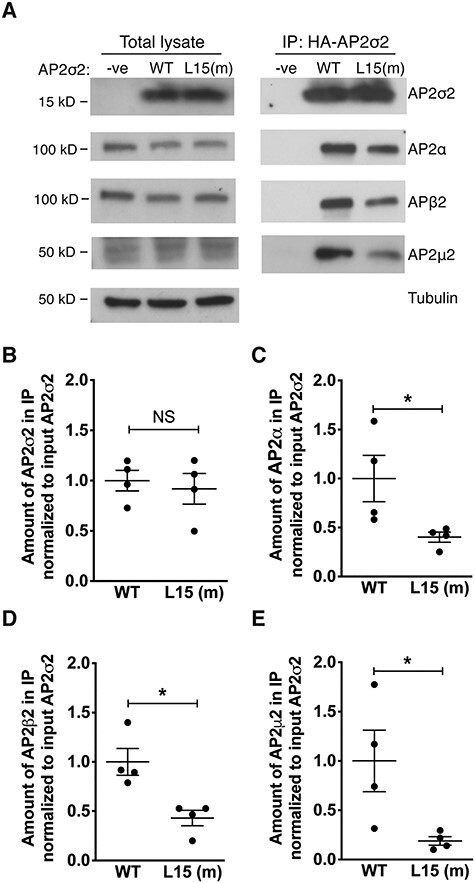
Co-immunoprecipitation analysis of the interaction between AP2σ2 and other subunits of the AP2 complex in HEK293 cells. (**A**) Immunoprecipitation using an anti-HA antibody and HEK293 cells stably expressing either: FLAG-tagged CaSR and HA-tagged WT AP2σ2 (WT); FLAG-tagged CaSR and HA-tagged mutant (m) Leu15 AP2σ2 (L15 (m)); or FLAG-tagged CaSR alone (-ve). The amount of protein in the total lysates and precipitated immune complexes (IP:HA-AP2σ2) was analyzed by western blotting using anti-HA, anti-AP2α, AP2β2, AP2μ2 and anti-tubulin antibodies. (**B**–**E**) Densitometry of the western blotting to quantify; (B) AP2σ2; (C) AP2α; (D) AP2β2 and (E) AP2μ2 in the immunoprecipitate of WT and L15 (m) cells, normalized to the amount of AP2σ2 in the total lysate (pre-normalized to tubulin). Mean ± SEM values are indicated, and data were analyzed using the Mann–Whitney *U* test. NS, non-significant; ^*^P < 0.05.

The AP2σ2 subunit interacts with the AP2α, AP2β2 and AP2μ2 subunits to form the heterotetrameric AP2 complex (24), and we therefore assessed the effects of the FHH3-associated p.Arg15Leu AP2σ2 mutation on the interactions with these other AP2 complex subunits. We undertook co-IP analysis using an anti-HA antibody and lysates from the WT (Arg15–AP2σ2–FlaC2) cells or mutant (Leu15–AP2σ2–FlaC2) cells, as these stably overexpress HA-tagged WT or mutant (Leu15) AP2σ2 proteins, and have endogenous expression of the AP2α, AP2β2 and AP2μ2 subunits (Fig. 6A). The amounts of AP2σ2 detected in the immunoprecipitate from WT (Arg15–AP2σ2–FlaC2) cells or mutant (Leu15–AP2σ2–FlaC2) cells were not significantly different (Fig. 6A and B), but significant reductions of >50% in the amount of endogenous AP2α, AP2β2 and AP2μ2 subunits were observed in the immunoprecipitate from mutant (Leu15–AP2σ2–FlaC2) cells compared with WT (Arg15–AP2σ2–FlaC2) cells (*P* < 0.05) (Fig. 6C–E). Thus, these findings indicate that the *AP2S1* FHH3-associated p.Arg15Leu mutation impairs the interaction between AP2σ2 and the other subunits of the AP2 heterotetrameric complex.

## Discussion

We have established by the use of CRISPR/Cas9 genome editing, a mouse model for FHH3, and this will enable the calcitropic roles of AP2σ2 and endosomal trafficking of the CaSR to be further evaluated together with pursuit of pathophysiological studies that are difficult to undertake in patients with this condition. Our results revealed that *Ap2s1^+/L15^* mice, which harbored a germline heterozygous *Ap2s1* p.Arg15Leu mutation, had a similar plasma biochemical phenotype to that reported for FHH3 patients, who have heterozygous loss-of-function *AP2S1* missense Arg15 mutations (p.Arg15Cys, p.Arg15His or p.Arg15Leu), and with those having the AP2σ2 p.Arg15Leu mutation being affected with the severest hypercalcaemia (3,7). Thus, *Ap2s1^+/L15^* mice had substantial hypercalcaemia with mean plasma calcium concentrations that were >0.5 mmol/l (>20%) above that of WT littermates (Table 2, Fig. 1). These findings contrast with other monogenic FHH mouse models; for example *Casr^+/−^* and *Gna11^+/−^* mutant mice, which are respective models for FHH1 and FHH2, typically have milder hypercalcaemia with plasma or serum calcium concentrations that are <10% above that of the WT values (19,25). The *Ap2s1^+/L15^* mice were also hypermagnesaemic, which is consistent with the phenotype of FHH3 patients (3). In addition, *Ap2s1^+/L15^* mice had significantly increased plasma PTH concentrations in association with hypophosphatemia (Table 2, Fig. 1), and this is in agreement with the findings from studies of two large FHH3 kindreds from Oklahoma (FHH_Ok_) and Northern Ireland (FHH_NI_), which have demonstrated that hypercalcemic family members, compared with normocalcaemic relatives, have significantly increased serum PTH concentrations with mild hypophosphataemia (4,26). Moreover, affected males and females from the FHH_Ok_ kindred were reported to have an age-related increase in PTH (4), and such an age-related increase in PTH was also observed in the female, but not male, *Ap2s1^+/L15^* mice (Supplementary Material, Table S2). The etiology of this age-related increase in PTH, which has not been reported in FHH1 and FHH2 kindreds, and basis of the gender differences remain to be elucidated. However, the increased plasma FGF23 concentrations that were observed in the *Ap2s1^+/L15^* mice (Table 2, Fig. 1) are likely to have a role in the etiology of hypophosphataemia. Thus, the increased plasma FGF23 concentrations, which are likely the result of elevations in PTH that promote osteoblast production of FGF23 (27), will act on the kidneys to increase excretion of phosphate that will lead to hypophosphataemia. Interestingly, genetically modified mice and patients with primary hyperparathyroidism are reported to have increased plasma FGF23 concentrations (28,29), although these have not been assessed in FHH patients to-date, and thus our findings from the *Ap2s1^+/L15^* mice indicate that such measurements of FGF23 are warranted in FHH patients. The increased plasma FGF23 concentrations would be expected to decrease 1,25-dihydroxyvitamin D synthesis in *Ap2s1^+/L15^* mice. However, mutant mice had no significant alterations in plasma 1,25-dihydroxyvitamin D (Table 2), and it is possible that such inhibitory effects of FGF23 were counteracted by an increase in PTH-mediated 1,25-dihydroxyvitamin D synthesis.

The calcitropic phenotype of *Ap2s1^+/L15^* mice showed some differences to FHH3 patients. Thus, only female *Ap2s1^+/L15^* mice were hypocalciuric, whereas male and female FHH3 patients have been reported to be hypocalciuric (urine calcium creatinine clearance ratio < 0.01) (3,4). In addition, *Ap2s1^+/L15^* mice showed no alterations in bone turnover or whole body BMD, whereas lumbar spine and/or femoral neck BMD has been reported to be decreased in ≥50% of FHH3 patients (3,9). Moreover, *Ap2s1^+/L15^* mice had a significant increase in ALP activity (Table 2, Fig. 1), which has not been observed in FHH3 patients (3). However, the increased circulating ALP activity in the *Ap2s1^+/L15^* mice was associated with normal plasma concentrations of the P1NP and CTX-1 bone turnover markers (Table 2), thereby indicating that the raised ALP of *Ap2s1^+/L15^* mice may be of extra-skeletal origin. The raised ALP of *Ap2s1^+/L15^* mice is unlikely to be of hepatic origin, as mice, in contrast to humans, express little or no ALP in the liver (30), and the increased ALP of *Ap2s1^+/L15^* mice may therefore possibly arise from a non-hepatic source such as the intestine.

AP2σ2 forms part of the heterotetrameric AP2 complex, which plays a pivotal role in clathrin-mediated endocytosis, and crystallographic studies have indicated that the WT Arg15 AP2σ2 residue is involved in binding to peptide sequences on membrane-associated cargo proteins, which contain acidic dileucine motifs such as that predicted to occur in the distal portion of the CaSR intracellular domain (15). We have previously proposed that substitution of the polar Arg15 residue with the FHH3-associated non-polar Leu15 residue would disrupt the interaction between AP2σ2 and this endocytic recognition motif of the plasma membrane-bound CaSR (7), and our co-IP studies using *Ap2s1^+/L15^* mouse kidneys and HEK293 cells stably overexpressing the AP2σ2 subunit, now provide the evidence for this specific AP2σ2–CaSR interaction and its impairment by the FHH3-associated mutant Leu15 AP2σ2 protein (Figs 4 and 5). Future studies exploring the impaired AP2σ2–CaSR interaction and also the interactions between AP2σ2 and other AP2 subunits, in parathyroid glands and kidneys using techniques such as proximity ligation assay may help to further elucidate the contribution of this altered interaction to the calcitropic phenotype of FHH3. Our findings also showed that cinacalcet does not rescue the impaired AP2σ2-CaSR interaction *in vitro*. Thus, cinacalcet may instead decrease plasma PTH and calcium concentrations of *Ap2s1^+/L15^* mice and FHH3 patients by increasing signaling responses from CaSRs expressed at the cell-surface and/or within endosomes of parathyroid cells (31). Moreover, cinacalcet may potentially act as a pharmacochaperone to promote anterograde trafficking of newly synthesized CaSRs from the endoplasmic reticulum to the cell-surface (31).

Our co-IP studies have also demonstrated that the FHH3-associated mutant Leu15 AP2σ2 impairs the interactions between AP2σ2 and the other subunits (AP2α, AP2β2 and AP2μ2) of the AP2 heterotetramer (Fig. 6), thereby highlighting the pivotal role of this mutation in disrupting clathrin-mediated endocytosis of cell-surface proteins. These findings suggest that *Ap2s1^+/L15^* mice (and FHH3 patients) may therefore exhibit non-calcitropic phenotypes in addition to the calcitropic abnormalities described above. Indeed, our investigations of the *Ap2s1^+/L15^* mice have revealed non-calcitropic biochemical features (Supplementary Material, Table S3). Most notably, *Ap2s1^+/L15^* females had significant reductions in both plasma total cholesterol concentration and HDL cholesterol, which is a major cholesterol fraction in mice (32). HDL cholesterol concentrations are regulated by the ATP binding cassette transporter A-1 (ABCA1), which promotes efflux of cellular cholesterol and mediates the formation of HDL particles (33), and ABCA1 mutations cause familial HDL deficiency (34). Cellular cholesterol efflux mediated by ABCA1 is influenced by clathrin-dependent and independent endocytic pathways (35), and this highlights the possibility that the AP2 complex may play a role in cholesterol efflux. Thus, the *Ap2s1* p.Arg15Leu mutation may potentially induce low plasma HDL cholesterol concentrations through effects on the ABCA1 protein, and further studies are required to elucidate this mechanism and also to assess whether FHH3 patients may have alterations of plasma lipid components. In FHH3 patients, non-calcitropic features such as neurodevelopmental disorders have been reported (3,5,6). Neurodevelopmental disorders are reported to affect >65% of FHH3 children, who may have mild to severe learning difficulties, and also behavioral disturbances such as autism-spectrum disorder (ASD) and attention deficit hyperactivity disorder (ADHD) (3,5,6). Our present study, which included juvenile and adult *Ap2s1^+/L15^* mice, did not detect any gross behavioral abnormalities, but specific behavioral, neurophysiological and cognitive assessments will be required to identify any occurrence of neurodevelopmental abnormalities in the *Ap2s1^+/L15^* mice.

*Ap2s1^L15/L15^* homozygotes, in contrast to *Ap2s1^+/L15^* mice, were sub-viable (Table 1), and most died within the early neonatal period. One possible explanation for this neonatal lethality is that the *Ap2s1^L15/L15^* mice developed neonatal severe hyperparathyroidism (NSHPT), similar to that reported for *Casr*^−/−^ mice (25). NSHPT is characterized by severe hypercalcaemia, skeletal demineralization and growth retardation, and *Casr*^−/−^ mice with NSHPT have been reported to die within 3–30 days after birth (25). However, most *Ap2s1^L15/L15^* homozygotes died earlier, typically within 48 h after birth, which may suggest an alternate etiology for their neonatal lethality, such as a generalized impairment of clathrin-mediated endocytosis caused by the disrupted interactions between mutant AP2σ2 and the other subunits of the AP2 heterotetrameric complex (Fig. 6C–E). Consistent with this, a missense mutation of another AP2 subunit, AP2μ2, has been reported to impair clathrin-mediated endocytosis, and to be associated with epilepsy and developmental encephalopathy (36). Two male *Ap2s1^L15/L15^* mice did survive into adulthood, and both had more severe hypercalcaemia than their male *Ap2s1^+/L15^* litter-mates (Supplementary Material, Table S4), thereby also indicating a dosage effect of the mutant Leu15 *Ap2s1* allele on plasma calcium concentrations.

Cinacalcet decreased plasma PTH and calcium concentrations in *Ap2s1^+/L15^* mice, such that a single dose caused a rapid and >50% decrease in plasma PTH concentrations and a substantially lowering of plasma calcium by ~0.40 mmol/l, although treated *Ap2s1^+/L15^* mice remained mildly hypercalcaemic (Figs 2 and 3). However, it is likely that longer term cinacalcet dosing would normalize plasma calcium concentrations in *Ap2s1^+/L15^* mice consistent with reports of cinacalcet-treated FHH3 patients (10,21,22). Of note, cinacalcet treatment resulted in hyperphosphataemia in WT mice (Fig. 3C), as has been previously described (19). In contrast, *Ap2s1^+/L15^* mice treated with a single dose of cinacalcet showed no increase in plasma phosphate (Fig. 3D). Potentially, the observed raised FGF23 concentrations of mutant mice (Fig. 1F) may have prevented elevations in plasma phosphate following cinacalcet treatment. Serum FGF23 has been reported to decrease at 24 h after cinacalcet treatment in rodents (37), and therefore longer term dosing studies will likely be required to evaluate the effect of this calcimimetic on FGF23 secretion and phosphate homeostasis in *Ap2s1^+/L15^* mice.

In summary, we have established a mouse model for FHH3, and shown that the germline p.Arg15Leu mutation affecting the ubiquitously expressed AP2σ2 protein leads to a predominantly calcitropic phenotype, most likely by impairing interaction of AP2σ2 with the CaSR. Moreover, we have demonstrated that cinacalcet has a rapid effect in decreasing plasma PTH concentrations and in alleviating the hypercalcaemia associated with FHH3.

## Materials and Methods

### Generation of Ap2s1^R15L^ mice

Mice harboring a c.G44T transversion (p.Arg15Leu) in the *Ap2s1* gene, which encodes the AP2σ2 protein, were generated by homology-directed repair using the CRISPR/Cas9 system, as reported (17,38). Single-guide RNAs (sgRNAs) targeting the genomic region encoding the Arg15 residue of AP2σ2 were designed (http://crispr.mit.edu/), with the sgRNA cutting nearest to the intended change taken forward (5′-3′: CCGGGCAGGCAAGACGCGCC, protospacer adjacent motif (PAM) sequence: TGG). A single stranded DNA oligo-deoxynucleotide (ssODN) donor template of 121 nt containing the c.G44T (p.Arg15Leu) point mutation together with a synonymous substitution, c.G48T (p.Leu16Leu), to protect the engineered allele from further re-processing by CRISPR/Cas9 reagents, was purchased as an Ultramer™ DNA oligonucleotide (IDT) with 4 phosphorothioate bonds at each 5′ and 3′ extremity (5′-3′: c.ACCTCCTCGATCAGCTTCTGCTTCTCGTCGTCATCGAACTGCATGTACCACTTGGCAAGGAGCGTCTTGCCTGCCCGGTTCTGGATAAGGATGAATCGGATCTAGAGCAAGCAGGGGAGGG). Cas9 mRNA (Tebu-Bio), sgRNA and ssODN were diluted and mixed in microinjection buffer (MIB; 10 mm Tris-HCl, 0.1 mm EDTA, 100 mm NaCl, pH 7.5) to the working concentrations of 100 ng/μl, 50 ng/μl each and 50 ng/μl, respectively, and micro-injected into the pronucleus of C57BL/6J zygotes, which were then implanted into three recipient CD1 dams. Founder mice harboring the targeted allele were identified by obtaining ear biopsy DNA, which was then amplified using the following primers (5′-3′): AGATGAACTAAAGCCTGGGGC and TGTTCTGTACGCAACGAGCC. Amplicons were analyzed by Sanger DNA sequencing and one mosaic founder mouse was mated with WT C57BL/6J mice to produce the F1 generation of heterozygous mice. The mutant allele was characterized in the F1 generation by using PCR, Sanger DNA sequencing and ddPCR copy counting using both a universal assay (Forward primer 5′-3′: TGCTTGCTCTAGATCCGATTCATC, Reverse primer 5′-3′: TCGTCGTCATCGAACTGCAT, Probe 5′-3′: CTTATCCAGAACCGGGCAGGCA) and a p.Arg15Leu mutant-specific assay (Forward primer 5′-3′: GCATGTACCACTTGGCAAGGA, Reverse primer 5′-3′: TGCTTGCTCTAGATCCGATTCATC, Probe 5′-3′: TCTTGCCTGCCCGGTTCTGGAT) to confirm that random donor integrations had not occurred. F1 heterozygous mice were then inter-crossed to generate the initial litters of WT, *Ap2s1^+/L15^* and *Ap2s1^L15/L15^* mice for assessment of viability. Subsequent generations of WT and *Ap2s1^+/L15^* mice were established by backcrossing *Ap2s1^+/L15^* mice onto the C57BL/6J strain background. All mice were kept in accordance with Home Office welfare guidance in an environment controlled for light (12 h light and dark cycle), temperature (21 ± 2°C) and humidity (55 ± 10%) at the Medical Research Council (MRC) Harwell Centre (39). Mice had free access to water (25 ppm chlorine) and were fed *ad libitum* on a commercial diet (RM3, Special Diet Services) that contained 1.24% calcium, 0.83% phosphorus and 2948 IU/kg of vitamin D (39). Animal studies were approved by the MRC Harwell Institute Ethical Review Committee, and were licensed under the Animal (Scientific Procedures) Act 1986, issued by the UK Government Home Office Department (PPL30/3271).

### Compounds

Cinacalcet (AMG-073 HCL) was obtained from Cambridge Bioscience (catalog no. CAY16042) and dissolved in DMSO or a 20% aqueous solution of 2-hydroxypropyl-β-cyclodextrin (Sigma-Aldrich, catalog no. H107), respectively, prior to use in *in vitro* and *in vivo* studies (19).

### Generation of stable cell lines

A pcDNA3 construct (Invitrogen) containing a full length human *CASR* cDNA (40) with an N-terminal FLAG tag (DYKDDDDK) was used to generate HEK293 cells stably expressing the CaSR with a N-terminal FLAG tag (FlaC cells), and maintained under G418 (geneticin) selection. Eight FlaC cell lines (FlaC1–8) were generated (Supplementary Material, Fig. S2A and B). Expression of the CaSR was confirmed by western blotting using anti-CASR (ADD, Abcam) and anti-FLAG antibodies (ab49763, Abcam) (Supplementary Material, Fig. S2A and B). SRE, NFAT and Fluo4-AM intracellular calcium mobilization assays were performed using methods previously described (41,42) in one of the cell lines—FlaC2 cells—to confirm a response to extracellular calcium stimulation (Supplementary Material, Fig. S2C), and therefore functionality of the CaSR signaling pathway. FlaC2 cells stably expressing WT (Arg15-AP2σ2-FlaC2) or mutant (Leu15-AP2σ2-FlaC2) AP2σ2 proteins were then generated using a pcDNA5 construct containing a full-length human *AP2S1* cDNA (16) with a C-terminal HA tag (YPYDVPDYA), and maintained under hygromycin and geneticin selection. Site-directed mutagenesis using the Q5 Site-Directed Mutagenesis kit (New England Biolabs) and *AP2S1* specific primers (Thermo Fisher Scientific) were used to generate the mutant (Leu15) *AP2S1* construct. Stably transfected WT Arg15–AP2σ2–FlaC2, mutant Leu15–AP2σ2–FlaC2, and control FlaC2 cells were cultured in DMEM media supplemented with 10% FCS. For drug compound studies, the cells were treated with 10 nm cinacalcet, or vehicle (DMSO), for 15 min prior to being washed with PBS and lysed for co-IP analysis.

### Co-immunoprecipitation and western blot analysis

Cells were lysed in ice-cold lysis buffer (0.5% NP40, 135 mm NaCl, 20 mm Tris pH 7.5, 1 mm EDTA, 1× protease inhibitor (Sigma-Aldrich) 2 mm Na_3_VO_4_, 10 mm NaF) and debris removed by centrifugation. Immunoprecipitations using anti-CASR antibodies were performed by mixing lysates with antibody for 30 min at 4°C prior to addition to protein G agarose beads (Cell Signalling Technology) and further mixing for 2 h. Alternatively, lysates were mixed with anti-FLAG-sepharose or anti-HA-sepharose beads (Cell Signalling Technology) and mixed for 2 h. Beads were then washed 5× with lysis buffer and proteins prepared in 4× Laemmli loading dye and resolved using 10% SDS-PAGE gel electrophoresis. Proteins were transferred to polyvinylidene difluoride membrane and probed with primary antibodies (CASR ADD, Abcam), AP2σ2 (ab92380, Abcam), FLAG (ab49763, Abcam), HA (CST3724, Cell Signalling Technologies), AP2α (BD610501, BD Biosciences), AP2β2 (BD610381, BD Biosciences), AP2μ2 (ab137727, Abcam), tubulin (ab15246, Abcam) and calnexin (AB2301, Merck), and HRP-conjugated secondary antibodies (715-035-150 and 711-035-152, Jackson ImmunoResearch), prior to visualization using Pierce ECL Western blotting substrate. Tubulin or calnexin protein expression was used as a loading control. Densitometry analysis was performed by calculating the number of pixels per band using ImageJ software.

### Collection of mouse kidneys for co-immunoprecipitation studies

Kidneys were collected from *Ap2s1^+/+^* and *Ap2s1^+/L15^* female mice, and snap frozen in liquid nitrogen, and subsequently stored at −80°C. The outer renal capsule was removed to access the cortex, which was dissected and lysed in ice-cold lysis buffer, as described above, for co-IP analysis.

### Plasma biochemistry and hormone analysis

Blood samples from juvenile mice (aged 8 weeks) and adult mice (aged 15–22 weeks) were collected from the lateral tail vein following application of topical local anesthesia for measurement of plasma PTH, or collected from the retro-orbital vein under isoflurane terminal anesthesia for measurement of other plasma biochemical parameters (39,43). Plasma was separated by centrifugation at 5000*g* for 10 min at 8°C, and analyzed for sodium, potassium, calcium, albumin, phosphate, magnesium, ALP activity, glucose, lipids, liver function tests, urea and creatinine on a Beckman Coulter AU680 analyzer, as described in (43). Plasma calcium was adjusted for variations in albumin concentrations using the formula: plasma calcium (mmol/l) − [(plasma albumin (g/l) − 30) × 0.02], as reported (39). Hormones were measured as follows: PTH using a two-site ELISA specific for mouse intact PTH (Immutopics, San Clemente, USA); 1,25-dihydroxyvitamin D by a two-step process involving purification by immunoextraction and quantification by enzyme immunoassay (Immunodiagnostic Systems); and intact FGF23 using a two-site ELISA kit (Kainos Laboratories), as described (19). C-terminal cross-linking telopeptide of type 1 collagen (CTX-1) was measured using a mouse-specific ELISA (Biorbyt Ltd). Procollagen type 1 N-terminal propeptide (P1NP) was measured by an enzyme immunoassay (EIA) (Immunodiagnostic Systems) (18).

### Metabolic cages and urine biochemistry analysis

Mice, aged 15–17 weeks, were individually housed in metabolic cages (Techniplast), and fed *ad libitum* on water and powdered chow. Mice were allowed to acclimatize to their environment over a 72 h period, as described, prior to collection of 24 h urine samples (19). Urine was analyzed for calcium, phosphate and creatinine on a Beckman Coulter AU680 analyzer (19). The fractional excretion of calcium and phosphate were calculated using the formula U*_x_*/P_x_^*^P_Cr_/U_Cr_, where U*_x_* is the urinary concentration of the filtered substance (substance *x*) in mmol/l, P*_x_* is the plasma concentration of substance *x* in mmol/l, U_Cr_ is the urinary concentration of creatinine in mmol/l and P_Cr_ is the plasma concentration of creatinine in mmol/l (19).

### Skeletal imaging

BMC and BMD were measured in mice, aged 12–16 weeks, by whole body DXA scanning, which was performed on mice anesthetized by inhaled isoflurane and using a Lunar Piximus densitometer (GE Medical Systems), as reported in (19). DXA images were analyzed using Piximus software (19).

### *In vivo* administration of cinacalcet

Mice, aged 14–22 weeks, were randomly allocated to receive cinacalcet or vehicle as a single oral gavage bolus (19). None of the mice had undergone any experimental procedures prior to dosing. Study investigators were blinded during animal handling and also when undertaking endpoint measurements. The primary experimental outcome was a change in plasma calcium at 2-h post-dose.

### Statistical analysis

All *in vitro* studies involved *n* = 3–4 biological replicates. Statistical analysis of *in vitro* data was undertaken using the Mann–Whitney *U* test for two group comparisons, or a two-way ANOVA with Bonferroni correction for multiple tests and *post-hoc* analysis. Mouse viability was assessed by binomial distribution analysis. One-way ANOVA followed by Sidak’s test for pairwise multiple comparisons were used for all *in vivo* analyses. Bartlett’s test was used to assess for unequal variances between groups, which were then log-transformed prior to one-way ANOVA. All analyses were performed using GraphPad Prism (GraphPad), and a value of *P* < 0.05 was considered significant for all analyses.

*Conflict of Interest statement*. None declared.

## Funding

Wellcome Trust Investigator Award (106995/Z/15/Z to R.V.T.); Wellcome Trust Clinical Training Fellowship (205011/Z/16/Z to V.J.S.).

## Supplementary Material

HMG-2020-D-00791_Hannan_Supplementary_Appendix_tracked_ddab076
